# Is VATS suitable for lung diseases with hemoptysis? Experience from a hemoptysis treatment center in China

**DOI:** 10.1186/s12890-023-02506-4

**Published:** 2023-06-14

**Authors:** Bing Wang, Li Yao, Jian Sheng, Xiaoyu Liu, Yuhui Jiang, Lei Shen, Feng Xu, Xiyong Dai

**Affiliations:** Department of Surgery, Wuhan Pulmonary Hospital, Baofeng Road No.28, Wuhan, Hubei China

**Keywords:** VATS, Hemoptysis, Lung disease, Complications, Treatment effectiveness

## Abstract

**Background:**

Surgery is crucial in the treatment of the potentially fatal pulmonary hemoptysis condition. Currently, most patients with hemoptysis are treated by traditional open surgery (OS). To illustrate the effectiveness of video-assisted thoracic surgery (VATS) for hemoptysis, we developed a retrospective study of surgical interventions for lung disease with hemoptysis.

**Methods:**

We collected and then analysed the data, including general information and post-operative outcomes, from 102 patients who underwent surgery for a variety of lung diseases with hemoptysis in our hospital between December 2018 and June 2022.

**Results:**

Sixty three cases underwent VATS and 39 cases underwent OS. 76.5% of patients were male (78/102). Comorbidities with diabetes and hypertension were 16.7% (17/102) and 15.7% (16/102) respectively. The diagnoses based on postoperative pathology included aspergilloma in 63 cases (61.8%), tuberculosis in 38 cases (37.4%) and bronchiectasis in 1 case (0.8%). 8 patients underwent wedge resection, 12 patients underwent segmentectomy, 73 patients underwent lobectomy and 9 patients underwent pneumonectomy. There were 23 cases of postoperative complications, of which 7 (30.4%) were in the VATS group, significantly fewer than 16 (69.6%) in the OS group (*p* = 0.001). The OS procedure was identified as the only independent risk factor for postoperative complications. The median (IQR) of postoperative drainage volume in the first 24 h was 400 (195–665) ml, which was 250 (130–500) ml of the VATS group, significantly less than the 550 (460–820) ml of the OS group (*p* < 0.05). The median (IQR) of pain scores 24 h after surgery was 5 (4–9). The median (IQR) of postoperative drainage tube removal time was 9.5 (6–17) days for all patients, and it was 7 (5–14) days for the VATS group, which was less than 15 (9–20) days for the OS group.

**Conclusion:**

VATS for patients with lung disease presenting with hemoptysis is an effective and safe option that may be preferred when the hemoptysis is uncomplicated and the patient's vital signs are stable.

## Introduction

Hemoptysis, specifically referring to expectoration of blood from the lower respiratory tract, can range from mild blood-streaked sputum, to massive, life-threatening frank blood [1, 2]. Recent studies have shown that aspergillus, tuberculosis (TB), cancer, and bronchiectasis are the most frequent etiologies of hemoptysis [3–5]. Various medical treatments, endobronchial methods of bleeding control methods, bronchial artery embolization (BAE), and surgery have been applied to control hemoptysis [6, 7]. Whereas modest blood in the sputum or minimal hemoptysis can usually be medically controlled, severe or recurrent hemoptysis that has failed to respond to medication may require interventional or surgical procedures. BAE controls bleeding by occluding the bleeding vessel, which is the most effective choice for treating life-threatening hemoptysis [8, 9]. However, patients who have undergone BAE remain at risk for developing recurrent hemoptysis, and surgery may be required as an emergent contingency if other methods fail [10, 11]. Surgery provides a definitive resolution by resecting the involved diseased lung tissue, encompassing the bleeding blood vessels. As the symptom of hemoptysis is evaluated, a diagnosis that requires surgical resection is frequently determined, and can be undertaken as both effective and safe treatment with acceptable morbidity and mortality. Previously, these surgical procedures were performed according to traditional open thoracic surgery (OS). However, with the development and further refinement of minimally invasive techniques, thoracoscopic surgery has become widely used and preferred for many of these procedures. For example, Yun JS et al. compared the OS and video-assisted thoracic surgery (VATS) in patients that presented with hemoptysis and found that VATS could reduce postoperative complications [12, 13].

With continued advancement in instrumentation and surgical experience, even more technically difficult thoracic operations have been successfully undertaken using minimally invasive surgical techniques such as VATS [14–16]. However, the proportion of VATS procedures in patients with hemoptysis remains in question and deserves further investigation. The intent of the present study was to retrospectively analyze patients who initially presented with hemoptysis in our hospital and subsequently underwent either VATS or OS during the previous eight years, specifically to compare the efficacy and safety of the two types of procedures.

## Materials and methods

### Ethics statement

This study was authorized by the Wuhan Pulmonary Hospital Ethics Committee (Approval No. (2023)01).

### Study design

We collected and retrospectively analyzed the medical data of 102 cases of lung diseases with hemoptysis who underwent pulmonary surgery in our hospital from December 2014 to June 2022. We recorded the basic demographic and medical information such as age, sex, comorbidities, BMI(Body Mass Index), HGB(haemoglobin), smoking, diagnosis, location of the pathology, preoperative BAE, and quantity of hemoptysis. Additionally, the surgical data, included the preoperative time, surgical incision, method of surgical resection, operative time duration, operative blood loss, postoperative drainage, postoperative complications, pain scores, postoperative hospitalization, postoperative duration of the pulmonary drainage tube, and hospitalization costs. Preoperative time was defined as the length of time from admission to surgery. Operative time was defined as the interval from skin incision to completion of skin suturing. The defined surgical procedures include wedge resection, segmentectomy, lobectomy, and pneumonectomy. The visual analogue scale (VAS) was used for measuring pain intensity. The VAS is a continuous scale comprised of a 10 cm horizontal line, whose extremes are labeled as "no pain" and "worst imaginable pain". Patients were requested to mark their level of pain on that line. Pain scores were recorded three times within 24 h following surgery, then averaged as the final pain score for analysis.

The data were divided into the VATS group and the OS group. We also divided the above data into two groups according to the presence or absence of complications.

### Patient selection (Fig. 1)

**Fig. 1:**
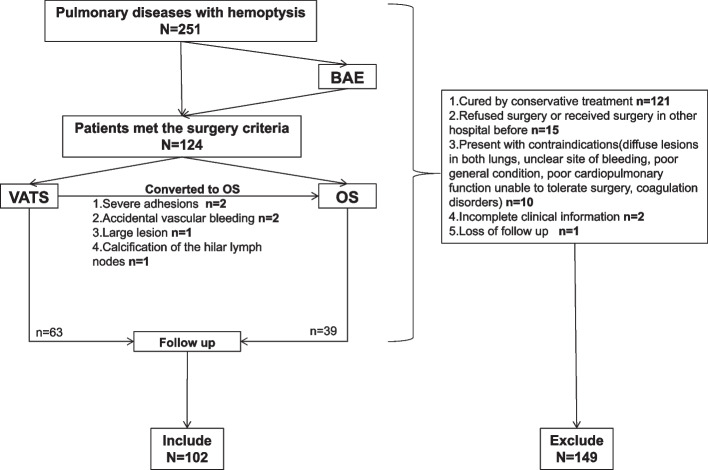
The flow chart of pulmonary diseases with hemoptysis in our hospital

Inclusion criteria were as follows:

1. Patients diagnosed with lung diseases with hemoptysis. 2. Patients underwent either VATS or OS in our hospital. 3. Complete clinical data were obtained. 4. Followed up for at least six months following surgery.

Exclusion criteria:

1. Patients refused surgery or had undergone pulmonary surgery in other hospitals. 2. Contraindications precluded surgical procedures, including diffuse lesions in both lungs, unclear site of bleeding, poor general condition, poor cardiopulmonary function unable to tolerate surgery, and coagulation disorders. 3. Impossible to complete following medication treatment. 4. Incomplete clinical information. 5. Loss of follow-up after surgery.

### BAE and surgical technique

Patients with hemoptysis were immediately assessed when admitted to our hospital. If the patient met an indication for BAE and consented for the procedure, BAE treatment was undertaken immediately. After BAE, the patient was re-evaluated and a decision was made regarding the need for surgery based on the patient's pathology and medical condition. If the patient was eligible for and had a surgical indication surgical treatment was performed immediately following patient consent. If a patient refused or was unable to undergo BAE but was eligible for surgery, we proceeded immediately after obtaining patient consent. Indications for surgery included: 1. irreversible lesions in a single lobe or in one lung; 2. a clear site of bleeding and stable vital signs, no cardiopulmonary failure, and no asphyxia; 3. patients with hemoptysis despite conservative treatment such as hemostatic drugs and BAE.

1. OS procedures: The patient was positioned on the operating table and prepped and draped in the usual sterile manner. A posterolateral thoracotomy incision of approximately 10–20 cm was made, usually either cutting one rib or removing a portion of a rib. Through the intercostal or costal bed, the thoracic cavity was entered and exposed with the use of a rib spreader. The diseased lung tissue was then systematically resected. In most cases, two chest tubes were inserted, the first into the 7th intercostal space, mid-axillary line, and the second in the third or fourth intercostal space in the mid-clavicular line.

2. VATS procedures: In like manner to OS procedures, the patient was positioned and prepped and draped in the usual manner. Initially, either a single 3–4 cm long incision, or three 1–3 cm long incisions are made in the intercostal space, and using ports through the incisions, a small camera and thoracoscopic surgical instruments were inserted to perform the surgery. There was no need to perform rib resection or rib spreader during VATS. The diseased lung tissue was systematically resected. Uni-portal VATS procedures typically ended with the placement of two chest tubes through the surgical incision, positioned crosswise on each side of the incision. Three-port VATS procedures involved placing one chest tube at the operating port, typically in the 4th or 5th intercostal space of the anterior axillary line, and another at the observation port, usually in the 7th intercostal space of the mid-axillary line.

3. Analgesic treatment: Every patient that underwent OS had a spinal paravertebral nerve block under ultrasound guidance by the anesthesiologist after anesthesia, and postoperatively, a standard analgesic pump (containing non-steroidal anti-inflammatory drugs) was routinely used for pain relief.

### Postoperative treatment and follow-up

We recorded all postoperative complications, such as air leakage, thoracic hemorrhage, pulmonary embolism and graded them according to the Clavien-Dindo criteria. Air leakage was defined as a persistent air leak from the chest tube and excluding a bronchopleural fistula in a lung wound that has not healed for 2 weeks after surgery. Postoperative thoracic hemorrhage was defined as active postoperative thoracic bleeding requiring intervention with drugs and other measures. Air leakage was evaluated using chest digital drainage equipment. The chest tube was typically removed when there was no pleura effusion, the drainage volume was less than 50 ml per day, there was no air leak from the chest bottle, and a chest X-ray indicated expansion of the lung. Each patient was followed for a minimum of six months. None of the patients had evidence of recurrence according to lung CT, bronchoscopy, sputum analysis, or symptoms.

### Statistical analysis

SPSS 22.0 (SPSS Inc, Chicago, IL) was used for the data analysis. Categorical variables were expressed in rates and percentages and compared using the χ2 test or Fisher's exact test. All continuous variables were analyzed for normal distribution with the Kolmogorov–Smirnov test and hospitalization costs were regularly dispersed distribution; other continuous variables in this study were irregularly dispersed distribution. We presented hospitalisation costs with Mean ± SD, used the T-test to compare them. The median (Interquartile Range) was used to characterize irregularly dispersed distribution continuous variables and used the Mann–Whitney U test for comparison. Preoperative basic information and intraoperative data were categorized by the presence or absence of complications with single-factor analysis; upon concluding that there were differences in the occurrence of complications in surgical incision and time of surgery, we then performed logistic regression analysis on the above differential data to determine the independent risk factors for postoperative complications. When the p-value was less than 0.05, the difference was statistically significant.

## Results

Table 1 shows the basic information, perioperative data, and postoperative complications of the 102 patients who were enrolled, 63 cases in the VATS group and 39 cases in the OS group. Six patients in the OS group were originally selected for VATS. Intraoperatively, two cases were converted to OS due to severe adhesions, two cases were due to sudden major vascular rupture and bleeding, one case was due to a large lesion, and one case was due to calcification of the hilar lymph nodes. The median (IQR) age of all patients was 55(47.5–62) years,and 76.5% of the patients were male (78/102). Comorbidities included diabetes, hypertension, liver disease, and immunological disease. The proportion of patients who smoked was 27.5%. Eight cases had a BMI lower than 18.5 kg/m^2^, and 3 cases were greater than 24 kg/m^2^*.* The median (IQR) haemoglobin for all patients was 114 (107–120). Three patients with hemoptysis were treated with blood transfusions before surgery. The diagnosis based on postoperative pathology included aspergilloma in 63 cases (61.8%), TB in 38 cases (37.4%), and bronchiectasis in 1 case (0.8%). Regarding lung disease location, 53.9% (55/102) were on the right and 46.1% (47/102) on the left. There were 14 cases (13.7%) that underwent preoperative BAE, and 19.6% (20/102) of the patients suffered from severe hemoptysis (> 100 mL/day). The Mean ± SD of hospitalization costs were 61,832 ± 6700RMB, there was no difference in hospitalization costs between the VATS and OS groups (*P* = 0.778). Hospitalization costs were higher in the group with complications than in the group without complications (*P* = 0.023).

**Table 1:** Basic information,perioperative information and Postoperative complications

**Variables**	**Patients** *N* = 102	**Surgical procedures**		P1	**Postoperative complications**		P2
		VATS *n* = 63	OS *n* = 39		Present *n* = 23	Absent *n* = 79	
**Age (years) Median (IQR)**	55(47.5–62)	55(48–63)	56(46–62)	0.945	58(48–62)	55(46–62)	0.614
**Gender (n, %)**							
* Male*	78(76.5%)	45(71.4%)	33(84.6%)	0.127	21(91.3%)	57(72.2%)	0.104
* Female*	24(23.5%)	18(28.6%)	6(15.4%)		2(8.7%)	22(27.8%)	
**Comorbidities*** (n, %)*							
* Diabetes*	17(16.7%)	12(19.0%)	5(12.8%)	0.412	3(13.0%)	14(17.7%)	0.832
* Hypertension*	16(15.7%)	9(14.3%)	7(17.9%)	0.621	1(4.3%)	15(19.0%)	0.170
* Liver diseases*	15(14.7%)	12(19.0%)	3(7.7%)	0.198	3(13.0%)	12(15.2%)	1.000
* Immunological diseases*	1(1%)	1(1.6%)	0	1.000	0	1(1.3%)	1.000
* Smoking (n,%)*	28(27.5%)	15(23.8%)	13(33.3%)	0.295	8(34.4%)	20(25.3%)	0.371
*BMI (n, %)*							
< *18.5 kg/m*^*2*^	8(7.8%)	5(7.9%)	3(7.7%)	0.228	2(8.7%)	6(7.6%)	0.445
* 18.5* ~ *23.9 kg/m*^*2*^	91(89.2%)	55(87.3%)	36(92.3%)		21(91.3%)	70(88.6%)	
≥ *24.0 kg/m*^*2*^	3(3.0%)	3(4.8%)	0		0	3(3.8%)	
* HGB(g/L)*Median(IQR)	114(107–120)	111(104–119)	115(110–121)	0.062	114(107–120)	114(105–120)	0.962
* Preoperative Blood Transfusion*	3(2.9%)	1(1.6%)	2(5.1%)	0.670	1(4.3%)	2(2.5%)	1.000
**Diagnosis (n, %)**							
* Pulmonary aspergilloma*	63(61.8%)	36(57.1%)	27(69.2%)	0.386	18(78.3%)	45(57.0%)	0.172
* Tuberculosis*	38(37.4%)	26(41.3%)	12(30.8%)		5(21.7%)	33(41.7%)	
* Bronchiectasia*	1(0.8%)	1(1.6%)	0		0	1(1.3%)	
**Location (n, %)**							
* Right*	55(53.9%)	37(58.7%)	18(46.2%)	0.216	13(56.5%)	42(53.2%)	0.776
* Left*	47(46.1%)	26(41.3%)	21(53.8%)		10(43.5%)	37(46.8%)	
**Preoperative BAE (n, %)**							
** Yes**	14(13.7%)	8(12.7%)	6(15.4%)	0.702	3(13.0%)	11(13.9%)	1.000
** No**	88(86.3%)	55(87.3%)	27(84.6%)		20(87.0%)	68(86.1%)	
**The amount of hemoptysis (n,%)**							
* Severe hemoptysis* ≥ *100 ml/day*	20(19.6%)	12(19.0%)	8(20.5%)	0.856	7(30.4%)	13(16.5%)	0.137
* Small hemoptysis* < *100 ml/day*	82(80.4%)	51(81.0%)	31(79.5%)		16(69.6%)	66(83.5%)	
**Hospitalization costs (RMB) Mean ± SD**	61,832 ± 6700	61,973 ± 6018	61,603 ± 7756	0.778	64,606 ± 8575	61,024 ± 5871	0.023^a^
**Preoperative time (hours) Median(IQR)**	15(10–21)	16(10–22)	12(8–20)	0.076	15(8–21)	10(8–24)	0.236
**Surgical resection method (n,%)**							
* Sublobar(wedge/segment)*	20(19.6%)	15(23.8%)	5(12.8%)	0.174	3(13.0%)	17(21.5%)	0.547
* Lobar/pneumonectomy*	82(80.4%)	48(76.2%)	34(87.2%)		20(87.0%)	62(78.5%)	
**Surgical procedures (n, %)**							
* VATS*					7(30.4%)	56(70.9%)	< 0.001^a^
* OS*					16(69.6%)	23(29.1%)	
**Operative time(minutes) Median(IQR)**	242.5(180–347.5)	210(150–290)	300(240–385)	0.000^a^	225(165–325)	320(245–395)	0.003^a^
**Operative blood loss(ml) Median(IQR)**	350(200–1000)	300(100–700)	500(300–1200)	0.011^a^	300(150–700)	600(300–1200)	0.154

^a^The difference was statistically significant

The median (IQR) preoperative time was 15(10–21) hours, which showed no statistically significant difference in the type of surgical procedure or postoperative complications (*p* > 0.05). 20 patients underwent sublobar (wedge or segmentectomy) resection, whereas 82 had either lobectomy or pneumonectomy. These aforementioned results showed no statistically significant differences between the VATS and the OS groups (*p* > 0.05). The data also indicate that there were no statistically significant differences in the variables between the groups with and without complications. However, there were variables that did show statistically significant differences comparing the two groups. The median (IQR) operative time of all patients was 242.5 (180–347.5) minutes, 210 (150–290) minutes for the VATS group, significantly less than the OS group at 300 (240–385) minutes (*p* < 0.05). The median (IQR) blood loss volume during the operation of all patients was 350 (200–1000) ml, 300 (100–700) ml for the VATS group, significantly less than the OS group at 500 (300–1200) ml (*p* = 0.011). The median (IQR) operative time of the group who subsequently experienced postoperative complications was 225 (165–325) minutes, significantly less than the 320 (245–395) minutes of the group who had not suffered complications (*p* < 0.003).

The type and timing of surgery were identified as risk factors by univariate analysis of risk factors for postoperative complications. According to multivariate logistical analysis, the OS procedures were verified as the only independent risk factor for postoperative complications. More details are given in Table 2.

**Table 2:** Results of multivariate analyses for postoperative complications

Variables	OR	95% CI	*P*
**Surgical procedures**	4.220	1.468–12.130	0.008*
**Operative time**	1.004	0.999–1.009	0.091

*OR* odds ratio, *CI* confidence interval

* The difference was statistically significant

Table 3 shows the postoperative details with VATS and OS. The median (IQR) postoperative drainage volume in the first 24 h for the VATS group was 400 (195–665) ml, which was significantly less than the OS group's 550 (460–820) ml (p 0.05). The median (IQR) postoperative drainage volume in the second 24 h was 220 (120–500) ml, which was 180 (100–300) ml in the VATS group and significantly less than the 500 (220–660) ml in the OS group (*p* < 0.05). The median (IQR) of postoperative drainage volume in 48 h was 700 (380–1012.5) ml, which was 500 (260–780) ml in the VATS group, significantly less than 1000 (760–1430) ml in the OS group (*p* < 0.05). In the 24 h following surgery, the median (IQR) pain score was 5 (4–9), which was significantly less than 10 (8–10) in the OS group (*p* < 0.05). The median (IQR) of hospital stay time after the operation were 13 (9–18.25) days, respectively; the VATS group were shorter than the OS group (*p* < 0.05). The median (IQR) postoperative drainage tube removal time was 9.5 (6–17) days for all patients; it was 7 (5–14) days for the VATS group, which was less than the 15 (9–20) days for the OS group.

**Table 3:** Postoperative details with VATS and OS

Postoperative Details	Patients *N* = 102	VATS *n* = 63	OS *n* = 39	*p*
Postoperative drainage volume in the first 24 h (ml) Median (IQR)	400(195–665)	250(130–500)	550(460–820)	< 0.001*
Postoperative drainage volume in the second 24 h (ml) Median (IQR)	220(120–500)	180(100–300)	500(220–660)	< 0.001*
Postoperative drainage volume in 48 h (ml) Median (IQR)	700(380–1012.5)	500(260–780)	1000(760–1430)	< 0.001*
Pain scores Median (IQR)	5(4–9)	4(4–5)	10(8–10)	< 0.001*
Hospital stay time after operation(days) Median (IQR)	13(9–18.25)	10(8–15)	18(13–21)	< 0.001*
Postoperative drainage tube removal time(days) Median (IQR)	9.5(6–17)	7(5–14)	15(9–20)	< 0.001*
Postoperative complications Present (n, %)	23(22.5%)	7(11.1%)	16(41.0%)	< 0.001*
Leakage (n, %)	16(15.7%)	5(7.9%)	11(28.2%)	0.006*
Haemorrge (n, %)	6(5.9%)	2(3.2%)	4(10.3%)	0.296
Pulmonary embolism (n, %)	1(1.0%)	0	1(2.6%)	0.382
Dead of myocardial infarction (n,%)	1(1.0%)	0	1(2.6%)	0.382

^*^The difference was statistically significant

### Postoperative complication and management

The overall incidence rate of postoperative complications was 22.5% (23/102), with Clavien-Dindo grade I complications in 1 patient, grade II in 17, grade IIIa in 5, and grade V in 1. Major postoperative complications included air leakage in 16 (grade I in 1, grade II in 12, IIIa in 3), postoperative hemorrhage in 6 patients (grade II in 4, IIIa in 2), pulmonary embolism in 1 (grade II), and myocardial infarction in 1 patient (grade V) [17]. In the VATS patients, complications developed in 11.1% (7/63), significantly lower than 41.0% (16/39) in the OS group (*p* < 0.001). Air leakage for greater than 14 days occurred in 16 cases, 7.9% (5/63)in the VATS group, in contrast to 28.2% (11/39; *p* = 0.006) in the OS group. Nevertheless, all cases of air leakage resolved with conservative measures such as medication, lung function exercises, and nutritional supplements, the medication we use for the treatment of air leaks are concentrations of 50% dextrose 50 ml or platelet-rich plasma 50 ml, which are usually administered by intrathoracic injection. One patient in the OS group suffered from both an air leak and pulmonary embolism but was successfully treated with conservative measures. Six patients experienced postoperative thoracic hemorrhage, two in the VATS group and four in the OS group. Two of these patients in the OS group required emergency surgery to control the bleeding; the other four were successfully managed with conservative treatments such as hemostatic medications and blood transfusions. A single patient in the OS group died of a postoperative myocardial infarction (Table 3).

## Discussion

Severe or persistent hemoptysis is a potentially life-threatening emergency with a high mortality rate that may result from hemodynamic instability, airway obstruction, or inadequate gas exchange. Usually, a pulmonary lesion involves a bronchial artery or less frequently, a branch of the pulmonary artery, which ruptures and results in hemoptysis. The bronchial vasculature is thought to be the source of severe hemoptysis in 90% of cases [18]. The most common causes of hemoptysis include malignancy, bronchiectasis, aspergillosis, and TB5. Notably, TB may be the leading cause of hemoptysis in regions with a high endemic burden of TB [19]. Survival of patients with severe hemoptysis is determined by the quantity of hemoptysis and the success of immediate interventions. Massive hemoptysis has been previously defined as more than 100-150 ml of expectorated blood in a 24-h period [20, 21]. The most common cause of death in patients with massive hemoptysis is asphyxia, which often prompts rapid intubation and restoration of a clear airway. Urgent control of hemoptysis may necessitate BAE with occlusion of the bleeding vessel, followed by management of the primary lung diseases [22, 23]. Because revascularization of the bleeding vessel after temporary hemostasis or BAE treatment is possible, definitive surgical resection of the primary lung disease must be considered once cardiopulmonary function is satisfactory and ensuring that there are no contraindications [24, 25].

Postoperative complications of pulmonary surgery undertaken for severe hemoptysis include thoracic hemorrhage, bronchopleural fistula, air leak, pulmonary embolism, atelectasis, and lung infection, with an incidence of approximately 25% [26]. Despite the need for emergent or urgent surgery, attention must be paid to minimizing postoperative complications. The current surgical options include OS and minimally invasive thoracoscopic procedures such as muti-VATS and uni-VATS. When compared to the elective surgical setting, OS is more traumatic, with more complications and slower recovery than minimally invasive procedures, which are less traumatic and associated with fewer complications and less postoperative pain. Additionally, as surgical proficiency has increased, these thoracoscopic techniques have shown markedly decreased operating time, reduced postoperative complications, lessening postoperative pain, and shortened hospitalization [27]. In this study of 102 patients, nearly two-thirds underwent VATS, yet the number of postoperative complications was less than half of those who underwent OS. These complications were not trivial, but fortunately only 1 had a fatal outcome. The majority of patients in this series had hemoptysis due to aspergillosis, followed by TB, and only one was diagnosed with bronchiectasis. We compared the differences between several diseases in terms of operative procedures and complications, none of which were statistically significant. In prior studies, complications often were directly related to the underlying disease process, with severe hemoptysis often resulting from bronchiectasis. The present study differed due to only a single patient with bronchiectasis, with the remaining patients diagnosed with either aspergillosis or TB. The CT appearance of these pulmonary infections and their eventual management were similar, so they had similar postoperative outcomes. Our study demonstrated that OS was a risk factor for the development of postoperative complications, consistent with the result of Yun et al. [12, 28]. The duration of surgery was closely related to the state of the disease and surgical proficiency, consistent with Lang and Liu et al., who reported that the time required for thoracoscopy was shorter than that of OS with the same underlying conditions [29, 30]. In this study, the operative time for the VATS group was shorter than the OS group. The quantity of intraoperative bleeding has reflected the degree of invasion of tissues and organs and has previously been directly correlated with the rate of postoperative complications [31]. In the present study, the amount of intraoperative bleeding in the VATS group was less than the OS group. Several factors impact the presence and severity of postoperative pain, but clearly, the length of incision, dissection or retraction of underlying muscles and soft tissue, and distraction or resection of ribs would be universally acknowledged as important. The thoracoscopic incision is small, with minimal distraction of underlying tissues from entering the pleural space. Not surprisingly, the short-term postoperative pain scores for patients in the VATS group were lower than the OS group, consistent with the results of current international studies [32, 33]. The time from surgery to postoperative chest tube removal depends predominantly on the recovery of the lungs. Again, the VATS group required less time to chest tube removal than the OS group, concordant with previous data [34]. Current studies have found that VATS reduces the length of postoperative hospitalization for surgical patients [35–37]. The present study mirrored these results, again favoring the VATS group. Counterbalancing the increased cost of surgical instruments in the VATS group, longer postoperative hospitalization, increased use of pain medication, and prolonged chest tube drainage contributed to higher hospital costs for the OS group. The treatment costs of patients in both the OS and VATS groups were collected, and there was no significant difference in hospital costs between the two groups. This is consistent with the results of other studies showing that thoracoscopic surgery does not increase the cost of hospitalization [38, 39]. However, when we grouped hospitalization costs by the presence or absence of complications, the costs were significantly higher in the group with complications than in the group without complications.

In the present study, there was no difference in the preoperative time between the different surgical procedures, nor was there a difference in the rate of postoperative complications with the preoperative time or whether they were embolized preoperatively. Current articles suggest that complications are more common in emergency surgery [10]. This may be related to the severity of the lesion and the patient's vital signs in emergency patients and may have little to do with the preoperative time. In addition, several patients in the OS group in this study were transferred from VATS to OS intraoperatively due to a large lesion blocking the view, sudden bleeding, or calcification of hilar lymph nodes. Therefore, the decision to perform minimally invasive surgery in patients with emergency or severe hemoptysis might initially depend on the patient's wishes together with surgical input, but a clear understanding by all that intraoperative circumstances might necessitate alteration of the original plans.

This study also has disadvantages. First, the sample size of this study is relatively small. Second, our study is a single-center retrospective study; the apparent advantages of VATS need to be further confirmed in subsequent prospective studies. Thirdly, there is almost certainly bias in the surgical management of our hemoptysis cases, with larger lesions, more intraoperative adhesions, and more trophoblastic vessels of the chest wall and hilum likely to be undertaken by OS.

In conclusion, VATS proved to be an effective and safe option for patients with lung disease presenting with hemoptysis. VATS did not increase overall hospital costs, appeared to reduce complications, shorten operative time, reduced intraoperative blood loss, reduced the volume of postoperative drainage, improved postoperative pain management, and reduced the length of postoperative hospital stay. Therefore, VATS may be preferred when hemoptysis has been controlled and the patient's vital signs are stable.

## Data Availability

All data are available from the corresponding author by email request.
